# *N*,*N*′,*N*′′-Tricyclo­hexyl­guanidinium iodide

**DOI:** 10.1107/S1600536811049683

**Published:** 2011-11-30

**Authors:** Farouq F. Said, Basem F. Ali, Darrin Richeson

**Affiliations:** a Department of Chemistry, Al al-Bayt University, Mafraq 25113, Jordan; b Department of Chemistry and Biochemistry, University of Ottawa, Ottawa, Ontario, Canada K1N 6N5

## Abstract

In the title compound, C_19_H_36_N_3_^+^·I^−^, the orientation of the cyclo­hexyl rings around the planar (sum of N—C—N angles = 360°) CN_3_^+^ unit produces steric hindrance around the N—H groups. As a consequence of this particular orientation of the tricyclo­hexyl­guanidinium cation (hereafter denoted CHGH^+^), hydrogen bonding is restricted to classical N—H⋯I and non-clasical (cyclo­hex­yl)C—H⋯I hydrogen bonds. The propeller CHGH^+^ cation and the oriented hydrogen-bonding interactions lead to a three-dimensional supra­molecular structure.

## Related literature

For background to guanidines, see: Ishikawa & Isobe (2002 ▶); Moroni *et al.* (2001 ▶); Yoshiizumi *et al.* (1998 ▶). The title salt is isomorphous with the chloride anion-analogue (Cai & Hu, 2006 ▶) and *N,N′,N′′*-triisopropyl­guanidinium chloride (Said *et al.*, 2005 ▶). (Ishikawa & Isobe, 2002 ▶). The structural features and hydrogen -bonding array provided by guanidinium cations suggest them to be good building blocks for the formation of supra­molecular entities, see: Said, Bazinet *et al.* (2006 ▶); Said, Ong *et al.* (2006 ▶). For bond-length data, see: Allen *et al.* (1987 ▶). 
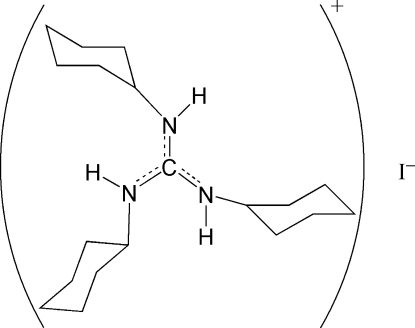


## Experimental

### 

#### Crystal data

C_19_H_36_N_3_^+^·I^−^
*M* *_r_* = 433.41
Cubic, 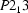

*a* = 12.893 (4) Å
*V* = 2143 (2) Å^3^
*Z* = 4
Mo *K*α radiation
μ = 1.50 mm^−1^
*T* = 188 K
0.5 × 0.3 × 0.3 mm

#### Data collection

Bruker P4 diffractometer
Absorption correction: multi-scan (*SADABS*; Bruker, 2005 ▶) *T*_min_ = 0.271, *T*_max_ = 0.320
2387 measured reflections
802 independent reflections
628 reflections with *I* > 2σ(*I*)
*R*_int_ = 0.055
3 standard reflections every 97 reflections  intensity decay: none

#### Refinement

*R*[*F* ^2^ > 2σ(*F* ^2^)] = 0.038
*wR*(*F* ^2^) = 0.076
*S* = 1.04
802 reflections
70 parameters
H-atom parameters constrained
Δρ_max_ = 0.33 e Å^−3^
Δρ_min_ = −0.27 e Å^−3^
Absolute structure: Flack (1983 ▶), 802 Friedel pairs
Flack parameter: 0.08 (8)

### 

Data collection: *XSCANS* (Bruker, 1996 ▶); cell refinement: *XSCANS*; data reduction: *XSCANS*; program(s) used to solve structure: *SHELXS97* (Sheldrick, 2008 ▶); program(s) used to refine structure: *SHELXL97* (Sheldrick, 2008 ▶); molecular graphics: *SHELXTL* (Sheldrick, 2008 ▶); software used to prepare material for publication: *SHELXTL*.

## Supplementary Material

Crystal structure: contains datablock(s) I, global. DOI: 10.1107/S1600536811049683/bq2321sup1.cif

Supplementary material file. DOI: 10.1107/S1600536811049683/bq2321Isup2.mol

Structure factors: contains datablock(s) I. DOI: 10.1107/S1600536811049683/bq2321Isup3.hkl

Supplementary material file. DOI: 10.1107/S1600536811049683/bq2321Isup4.cml

Additional supplementary materials:  crystallographic information; 3D view; checkCIF report

## Figures and Tables

**Table 1:** Hydrogen-bond geometry (Å, °)

*D*—H⋯*A*	*D*—H	H⋯*A*	*D*⋯*A*	*D*—H⋯*A*
N1—H1*A*⋯I1^i^	0.86	2.86	3.693 (5)	165
C2—H2*A*⋯I1^ii^	0.98	3.03	3.950 (5)	158

Symmetry codes: (i) 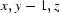; (ii) 
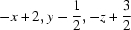
.

**Fig. 1.**
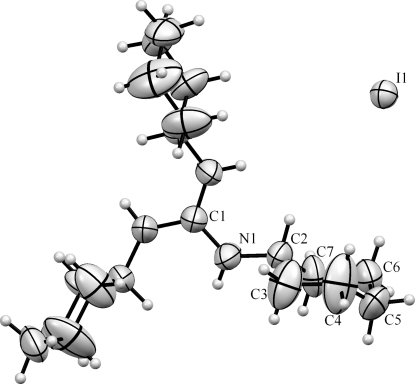
The structure of (I) with the guanidinium cation symmetry unique atoms are labeled. The other atoms are related by threefold rotation (3/2 – z, 1 – x, 1/2 + y and 1 – y, – 1/2 + y, 3/2 – x).

**Fig. 2.**
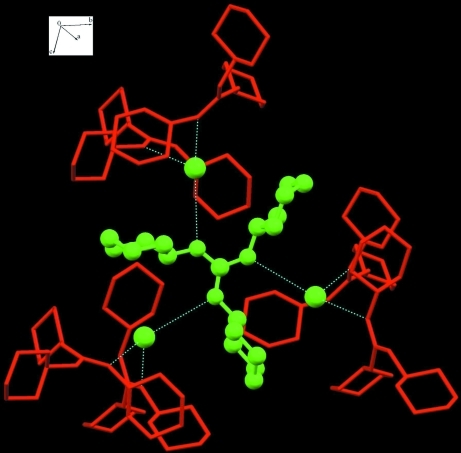
A partial packing diagram of (I), showing the CHGH+ cations and anions occur in a 3-fold array: three anions surround each cation and three cations surround each anion. Different colors and molecular rendering is used to clarify the arrangement.

**Fig. 3.**
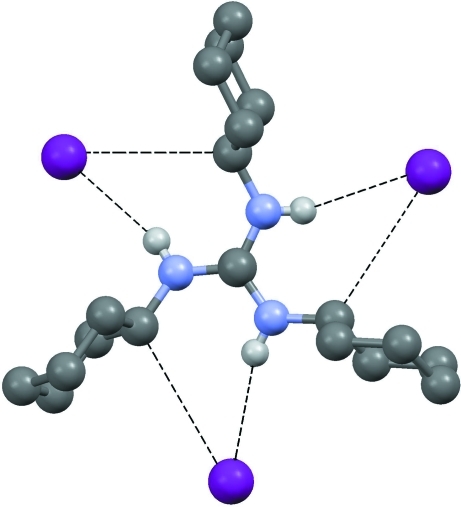
The diagram showing one guanidium cation and three anions in order to emphasize the orientation of the supramolecular synthon that results from hydrogen bonding array of three N—H···I and three C—H···I interactions.

## supplementary crystallographic information

### Comment

Guanidines are of special interest due to their possible application in medicine (Yoshiizumi *et al.*, 1998; Moroni *et al.*, 2001). They are considered super bases as they are easily protonated to generate guanidinium cations (Ishikawa & Isobe, 2002). The structural features and hydrogen bonding array provided by these cations suggest that they are good building blocks for the formation of supramolecular entities (Said, Bazinet *et al.*, 2006, Said, Ong *et al.*, 2006, Said *et al.*, 2005).

The title compound (I), Fig. 1, is a typical *N*,*N'*,*N"*-trisubstituted guanidinium halide salt with normal geometric parameters (Said *et al.*, 2005). The central guanidinium fragment of the cation of the title salt is planar [sum of NCN angles is 360°] with bond lengths and angles as expected for a central C*sp*^2^ hybridization, accounting for charge delocalization between the three C—N bonds. The bond length C1—N1 [1.330 (5) Å] is comparable with literature averages for substituted and unsubstituted guanidinium cations (1.321 and 1.328 Å, respectively; (Allen *et al.*, 1987)). The cyclohexyl ring has the normal chair conformation with conventional bond lengths and angles. A partial packing diagram is shown in Fig. 2. The CHGH^+^ ions occur in chains, with the I^-^ anions arranged parallel to the cation chains. The cations and anions occur in a 3-fold array: three anions surround each cation [*via* its three N—H···I, 2.856 Å; (165°) and C—H···I (3.027 Å; 158°) interactions, Table 1, Fig. 3], and three cations surround each anion resulting in the formation of three-dimensional supramolecular structure.This type of supramolecular synthons has been observed frequently in other related compounds. The stability of this crystal lattice is evidenced by the crystallization of a whole series of isomorphous compounds of this type, such as *N,N',N''*-tricyclohexylguanidinium chloride (Cai & Hu, 2006), even with different substituents like *N,N',N''*-triisipropylguanidinium chloride (Said *et al.*, 2005).

### Experimental

General:*N,N^'^,N^"^-*tricyclohexylguanidine was prepared according to literature methods. All other reagents were purchased from Aldrich Chemical Company and used without further purification. Elemental analyses were run on a Perkin Elmer PE CHN 4000 elemental analysis system.

Synthesis and crystallization of*N,N',N"-*tricyclohexylguanidinium iodide, {*C*(HNcyclohexyl)_3_}^+^I^-^

In a round bottom flask, a combination of 0.200 g (1.34 mmol) ammonium iodide and 0.41 g (1.34 mmol) *N,N^'^,N^''^*-tricyclohexylguanidine were dissolved in 10 mL of distilled water. White precipitate of {*C*(HNcyclohexyl)_3_}^+^I^-^ was deposited immediately of the solution (0.46 g, 92.0% yield). The product was crystallized from a mixture of methanol and distilled water to give white cubic crystals. In addition to confirming the molecular formula through elemental analysis, the solid obtained was examined by single-crystal X-ray analysis. Anal. Calcd for C_19_H_36_IN_3_ C, 52.65; H, 8.37; N, 9.70. Found C, 52.56; H, 8.63; N, 9.40.

### Refinement

Hydrogen atoms were included in calculated positions and refined as riding on their parent atoms with C—H = 0.97 Å and *U*_iso_(H) = 1.2*U*_eq_(C) and N—H = 0.86 Å and *U*_iso_(H) = 1.2*U*_eq_(N).

### Crystal data

**Table:**

C_19_H_36_N_3_^+^·I^−^	*D*_x_ = 1.343 Mg m^−^^3^
*M**_r_* = 433.41	Mo *K*α radiation, λ = 0.71073 Å
Cubic, *P*2_1_3	Cell parameters from 30 reflections
Hall symbol: P 2ac 2ab 3	θ = 3.9–6.9°
*a* = 12.893 (4) Å	µ = 1.50 mm^−^^1^
*V* = 2143 (2) Å^3^	*T* = 188 K
*Z* = 4	Block, colorless
*F*(000) = 896	0.5 × 0.3 × 0.3 mm

### Data collection

**Table:**

Bruker P4 diffractometer	628 reflections with *I* > 2σ(*I*)
Radiation source: fine-focus sealed tube	*R*_int_ = 0.055
graphite	θ_max_ = 25.9°, θ_min_ = 2.2°
ω scans	*h* = 0→15
Absorption correction: multi-scan (*SADABS*; Bruker, 2005)	*k* = 0→15
*T*_min_ = 0.271, *T*_max_ = 0.320	*l* = 0→15
2387 measured reflections	3 standard reflections every 97 reflections
802 independent reflections	intensity decay: none

### Refinement

**Table:**

Refinement on *F*^2^	Secondary atom site location: difference Fourier map
Least-squares matrix: full	Hydrogen site location: inferred from neighbouring sites
*R*[*F*^2^ > 2σ(*F*^2^)] = 0.038	H-atom parameters constrained
*wR*(*F*^2^) = 0.076	*w* = 1/[σ^2^(*F*_o_^2^) + (0.0269*P*)^2^ + 0.7683*P*] where *P* = (*F*_o_^2^ + 2*F*_c_^2^)/3
*S* = 1.04	(Δ/σ)_max_ < 0.001
802 reflections	Δρ_max_ = 0.33 e Å^−^^3^
70 parameters	Δρ_min_ = −0.27 e Å^−^^3^
0 restraints	Absolute structure: Flack (1983), 802 Friedel pairs
Primary atom site location: structure-invariant direct methods	Flack parameter: 0.08 (8)

### Special details

**Table:**

Geometry. All e.s.d.'s (except the e.s.d. in the dihedral angle between two l.s. planes) are estimated using the full covariance matrix. The cell e.s.d.'s are taken into account individually in the estimation of e.s.d.'s in distances, angles and torsion angles; correlations between e.s.d.'s in cell parameters are only used when they are defined by crystal symmetry. An approximate (isotropic) treatment of cell e.s.d.'s is used for estimating e.s.d.'s involving l.s. planes.
Refinement. Refinement of *F*^2^ against ALL reflections. The weighted *R*-factor *wR* and goodness of fit *S* are based on *F*^2^, conventional *R*-factors *R* are based on *F*, with *F* set to zero for negative *F*^2^. The threshold expression of *F*^2^ > σ(*F*^2^) is used only for calculating *R*-factors(gt) *etc*. and is not relevant to the choice of reflections for refinement. *R*-factors based on *F*^2^ are statistically about twice as large as those based on *F*, and *R*- factors based on ALL data will be even larger.

### Fractional atomic coordinates and isotropic or equivalent isotropic displacement parameters (Å^2^)

**Table:**

	*x*	*y*	*z*	*U*_iso_*/*U*_eq_	
I1	0.89107 (4)	0.89107 (4)	0.89107 (4)	0.0589 (2)	
N1	0.8894 (5)	0.1398 (4)	0.7489 (4)	0.0715 (16)	
H1A	0.8803	0.0781	0.7728	0.086*	
C1	0.8343 (6)	0.1657 (6)	0.6657 (6)	0.064 (3)	
C2	0.9631 (6)	0.2045 (6)	0.8033 (6)	0.071 (2)	
H2A	0.9812	0.2636	0.7591	0.086*	
C3	0.9164 (6)	0.2440 (8)	0.9017 (8)	0.114 (4)	
H3A	0.8951	0.1861	0.9448	0.137*	
H3B	0.8556	0.2855	0.8862	0.137*	
C4	0.9957 (9)	0.3091 (9)	0.9588 (11)	0.149 (5)	
H4A	1.0115	0.3701	0.9177	0.179*	
H4B	0.9660	0.3323	1.0239	0.179*	
C5	1.0919 (7)	0.2525 (9)	0.9799 (7)	0.100 (3)	
H5A	1.0780	0.1967	1.0285	0.120*	
H5B	1.1419	0.2990	1.0116	0.120*	
C6	1.1359 (5)	0.2091 (7)	0.8838 (7)	0.084 (2)	
H6A	1.1957	0.1668	0.9009	0.101*	
H6C	1.1593	0.2654	0.8397	0.101*	
C7	1.0581 (6)	0.1441 (7)	0.8255 (7)	0.086 (3)	
H7C	1.0885	0.1206	0.7608	0.103*	
H7A	1.0404	0.0835	0.8663	0.103*	

### Atomic displacement parameters (Å^2^)

**Table:**

	*U*^11^	*U*^22^	*U*^33^	*U*^12^	*U*^13^	*U*^23^
I1	0.0589 (2)	0.0589 (2)	0.0589 (2)	−0.0007 (3)	−0.0007 (3)	−0.0007 (3)
N1	0.084 (4)	0.060 (3)	0.070 (4)	−0.014 (4)	−0.026 (4)	0.014 (3)
C1	0.064 (3)	0.064 (3)	0.064 (3)	−0.012 (4)	−0.012 (4)	0.012 (4)
C2	0.082 (6)	0.071 (5)	0.061 (5)	−0.020 (5)	−0.024 (4)	0.010 (4)
C3	0.082 (7)	0.132 (8)	0.129 (9)	0.040 (6)	−0.022 (7)	−0.045 (8)
C4	0.129 (9)	0.147 (10)	0.172 (12)	0.014 (9)	−0.035 (9)	−0.102 (10)
C5	0.097 (7)	0.138 (8)	0.065 (5)	−0.021 (8)	−0.028 (6)	0.008 (6)
C6	0.064 (5)	0.090 (6)	0.098 (6)	−0.014 (4)	−0.005 (5)	0.007 (6)
C7	0.049 (4)	0.104 (7)	0.105 (6)	−0.010 (4)	−0.001 (5)	−0.026 (6)

### Geometric parameters (Å, °)

**Table:**

N1—C1	1.330 (5)	C4—C5	1.465 (13)
N1—C2	1.446 (9)	C4—H4A	0.9700
N1—H1A	0.8600	C4—H4B	0.9700
C1—N1^i^	1.330 (5)	C5—C6	1.473 (13)
C1—N1^ii^	1.330 (5)	C5—H5A	0.9700
C2—C7	1.479 (10)	C5—H5B	0.9700
C2—C3	1.493 (11)	C6—C7	1.508 (10)
C2—H2A	0.9800	C6—H6A	0.9700
C3—C4	1.514 (13)	C6—H6C	0.9700
C3—H3A	0.9700	C7—H7C	0.9700
C3—H3B	0.9700	C7—H7A	0.9700
			
C1—N1—C2	126.7 (5)	C5—C4—H4B	109.1
C1—N1—H1A	116.7	C3—C4—H4B	109.1
C2—N1—H1A	116.7	H4A—C4—H4B	107.8
N1^i^—C1—N1	119.99 (3)	C4—C5—C6	111.1 (8)
N1^i^—C1—N1^ii^	119.99 (3)	C4—C5—H5A	109.4
N1—C1—N1^ii^	119.99 (3)	C6—C5—H5A	109.4
N1—C2—C7	109.5 (6)	C4—C5—H5B	109.4
N1—C2—C3	110.1 (7)	C6—C5—H5B	109.4
C7—C2—C3	110.4 (7)	H5A—C5—H5B	108.0
N1—C2—H2A	108.9	C5—C6—C7	112.0 (7)
C7—C2—H2A	108.9	C5—C6—H6A	109.2
C3—C2—H2A	108.9	C7—C6—H6A	109.2
C2—C3—C4	109.3 (8)	C5—C6—H6C	109.2
C2—C3—H3A	109.8	C7—C6—H6C	109.2
C4—C3—H3A	109.8	H6A—C6—H6C	107.9
C2—C3—H3B	109.8	C2—C7—C6	110.8 (7)
C4—C3—H3B	109.8	C2—C7—H7C	109.5
H3A—C3—H3B	108.3	C6—C7—H7C	109.5
C5—C4—C3	112.7 (8)	C2—C7—H7A	109.5
C5—C4—H4A	109.1	C6—C7—H7A	109.5
C3—C4—H4A	109.1	H7C—C7—H7A	108.1

Symmetry codes: (i) −*z*+3/2, −*x*+1, *y*+1/2; (ii) −*y*+1, *z*−1/2, −*x*+3/2.

### Hydrogen-bond geometry (Å, °)

**Table:**

*D*—H···*A*	*D*—H	H···*A*	*D*···*A*	*D*—H···*A*
N1—H1A···I1^iii^	0.86	2.86	3.693 (5)	165
C2—H2A···I1^iv^	0.98	3.03	3.950 (5)	158

Symmetry codes: (iii) *x*, *y*−1, *z*; (iv) −*x*+2, *y*−1/2, −*z*+3/2.
